# Turning over New Leaves

**Published:** 1888-10-13

**Authors:** 


### Turning Over New Leaves.

[Books for Review should be sent to The Editor, The Lodge, Porchester Square, W.]

### * Sketches of Hospital Life.

This is an interesting and useful little book with a modest name. On the very first page Miss Morten dispels an almost universal illusion, which has been productive of a great deal of disappointment and suffering to a good many people. The "born nurse," she declares "is a myth : we may wait for a considerable time before making the acquaintance of a woman who came into the world possessed of a knowledge of the clinical thermometer, or the system of reversing a roller bandage. The glamour of romance which has been thrown round the service of the sick is being rapidly dispelled in these practical days. Nevertheless many sentimental or wayward girls (most appropriate description) embrace this calling, having no idea of the hard physical work and mental strain which await them. The old idea that nursing consisted chiefly in smoothing pillows, holding the cup of drink to the fevered lips, or reading to a partially convalescent patient, fades but slowly, and many a probationer discovers, to her disgust that she has undertaken a task from which the strongest man might shrink." These words are truth itself.

A case occurs to the mind of the writer, of a young lady, tenderly brought up, though almost without education and discipline, who four or five years ago resolved to sacrifice her youth and loveliness on the altar of nursing. She entered at ——, where she remained a short time, and then left for some apparently sufficient reason. From —— she went to ——, and from —— to —— again ; from High Church to Evangelical, and from Evangelical to Unsectarian institutions ; from London to the country, and from the country to the Colonies ; from general to children's hospitals, and back again to the general. She is now, or was a few days ago, trying her ninth or tenth institution. She is no more fit for nursing than she is for soldiering or ploughing. But her friends are still deluded with the idea that she is a "born nurse."

Miss Morten's little book consists of half-a-dozen not very long chapters. Nurses will enjoy it, and if they are sensible, will profit by it. Those who are not yet nurses, but who have cast longing eyes on the nursing field, should make a point of reading it. The price is only a shilling. The outlay may prove to be the saving, not only of many shillings, but of many disappointments and heartbreaks.

* By Honnor Morten : London : Sampson Low, Marston, Searle, and Rivington (Limited). Price One Shilling.

